# Effectiveness of education on radiation exposure for medical students prior to clinical clerkship

**DOI:** 10.1007/s11604-025-01815-4

**Published:** 2025-06-21

**Authors:** Satoshi Yamauchi, Kaede Kido, Ryosuke Taiji, Tomoko Ochi, Chisa Yoneima, Yuto Chanoki, Keisuke Oshima, Kohei Wakatsuki, Toshihiro Tanaka

**Affiliations:** 1 Department of Diagnostic and Interventional Radiology, Nara Medical University, Shijo Cho 840 Kashihara city, Nara, Japan; 2 Education Development Center, Nara Medical University, Shijo Cho 840 Kashihara city, Nara, Japan

**Keywords:** Radiation exposure, Medical student, Education, Clinical clerkship

## Abstract

**Purpose:**

The widespread use of radiation in diagnostic imaging, interventional procedures, and radiotherapy requires robust radiation safety education. Despite its importance, medical students in Japan and other countries have limited awareness of radiation protection. In Japan, radiation safety education is essential due to recent legislative changes that position medical students as “student doctors” and expand their clinical responsibilities. The purpose of this study was to evaluate the knowledge, concerns, and anxieties of 5th-year medical students regarding radiation exposure before clinical training, and to evaluate the impact of a lecture-style intervention on these parameters.

**Materials and methods:**

A 20-min lecture on radiation safety was given to 95 fifth-year students at Nara Medical University immediately before clinical training. The lecture covered the basic concepts of radiation biology, legal regulations, radiation risks, and protective measures. A 25-item questionnaire using a five-point Likert scale was administered before and after the lecture. Exploratory factor analysis was conducted on the data, and mixed-design analysis of variance (ANOVA) was used to evaluate changes in the main factors identified by exploratory factor analysis. Subgroup analysis was also conducted based on gender and the selected clinical department.

**Results:**

Exploratory factor analysis identified four main factors: anxiety, interest, knowledge, and management of radiation exposure. After the lecture-based intervention, knowledge and management of radiation exposure scores increased significantly (*p* < .001), but interest did not change. There was no difference in score by gender.

**Conclusion:**

Lecture-based interventions can significantly improve senior medical students’ knowledge and management skills regarding radiation safety. However, it was difficult to improve the interest of students who chose departments that were considered to have a low risk of radiation exposure. Our findings indicate the need for further development of educational strategies to improve awareness and education of radiation protection in clinical situations.

## Introduction

The history of the relationship between medicine and radiation is long, and its importance in modern medicine is growing. For example, in diagnostic imaging (e.g., X-ray and CT), interventional radiology, and radiation therapy for malignant tumors, the applications of radiation in medical practice are highly diverse, and have increasingly become a focus in education [1]. There is no doubt that the use of radiation in medical treatment has contributed greatly to the development of medicine, but it is also widely recognized and accepted that radiation exposure can increase the risk of various cancers [2]. Exposure to radiation in medical settings is more common in developed countries and high-income regions, and less so in emerging economies [3]; however, as the world continues to develop economically, interest in radiation exposure in medical settings is expected to grow, and the importance of education in this area will likewise increase worldwide. The health effects of radiation exposure are especially significant for children, and in developing countries this issue cannot be ignored [4].

For this reason, education on radiation exposure for medical students, who will be using these techniques in their future practice, is extremely important, but until now it has not been given much emphasis in Japan or other developed countries. Previous global studies have shown that there is still a low level of understanding of radiation protection among medical students [5–8]. This situation can lead to unsafe medical practices, potentially increasing the risk of unnecessary exposure for both students and patients.

Until recently, education for higher-year students at Japanese medical schools has mainly involved visits to clinical sites. It is common for training in actual medical practice to begin after becoming a licensed doctor. The same trend is seen at Nara Medical University, where lectures and other activities provide general knowledge about radiation exposure, yet practical education on radiation protection during training is minimal.

However, in 2023, the law regarding doctors in Japan was revised, and medical students who pass the public examinations (Computer-Based Testing (CBT) and Objective Structured Clinical Examination (OSCE)) are now called “Student Doctors” and are strongly encouraged to actively perform medical procedures and duties as part of the medical team. The risk of medical students being exposed to radiation during clinical clerkship has also increased significantly, and in the core curriculum, the importance of managing radiation exposure during such training, which had not been emphasized until recently, has been clearly stated. We have also begun lending electronic radiation dosimeters to students and recording their radiation doses. At the same time, there is a growing need to re-confirm knowledge of radiation exposure, radiation protection techniques, and methods for managing radiation exposure prior to clinical clerkship [9].

Although upper-year medical students have studied radiation to some extent, practical training in scenarios involving radiation exposure is rare, leaving it unclear how aware they are of radiation protection or how much fear they harbor regarding exposure. There is also a need to ensure patient safety with respect to medical radiation exposure, yet this aspect has received little attention. In fact, Faggioni et al. [10] reported that medical students were much less interested in radiation exposure than radiographers. Thus far, research reports on radiation exposure education for nuclear power plant workers [11], nurses [12] and high school students [13] in Japan following the Fukushima accident have been confirmed, but no studies have verified the effectiveness of practical educational methods for medical students.

The aims of this study are to assess the knowledge, concerns, and anxieties of medical students regarding radiation exposure before clinical practice. We will also clarify whether differences exist between genders and among clinical departments chosen by students, since the maximum allowed radiation exposure differs by gender and certain departments (such as cardiology and radiology) entail a high exposure risk. We will also evaluate the impact of lecture-style educational interventions.

## Materials and methods

### Course design

The lecture was delivered to 95 medical students (70 males, 25 females/average age 22.5 y.o.) at Nara Medical University just before their clinical clerkship. Owing to room constraints, the students were divided into two groups of approximately 50 each, and the same lecture was delivered to each group in a brief session of about 20 min. The lecture was presented using PowerPoint slides and included opportunities for brief questions and answers. The lecturer was a board-certified radiologist with extensive experience in radiation safety.

The lecture was designed to help students avoid potential issues during the clinical training program commencing the following week. The specific content of the lecture was as follows: the need to learn about radiation exposure, a review of basic biological knowledge about radiation, Japanese legal regulations regarding radiation exposure, the dangers of radiation exposure, and how to protect oneself from radiation. The lecture contents included visual aids such as diagrams and charts illustrating radiation principles and safety measures. They had previously received education on a similar topic over a year ago as part of their radiology course, so this lecture was a review and further development of that, so it was not completely first-time learning.

### Questionnaire design

The same 25-item questionnaire was used before and after the lecture on radiation exposure. The questionnaire was designed to comprehensively assess anxiety regarding radiation exposure, the degree of fear, and the confidence in specific responses, using a five-point Likert scale (strongly agree, somewhat agree, neither agree nor disagree, somewhat disagree, strongly disagree). In composing the scale for radiation exposure, we modified the items in the Schraw and Dennison [14] metacognition scale to obtain knowledge and interest in radiation exposure within individuals. In addition to these, we also created items based on the factors that cause anxiety about radiation exposure [15], with references to the study by Fukasawa et al. [16]on the Fukushima nuclear power plant accident, to obtain information about anxiety about radiation exposure (see Table 1 for details). The survey was conducted immediately before and after the lecture. Students accessed the designated URL via their smartphones, and responses were collected on the same day. The web survey requesting responses before the lecture is referred to as the pre-survey, and the web survey requesting responses after the lecture is referred to as the post-survey.

**Table 1:**
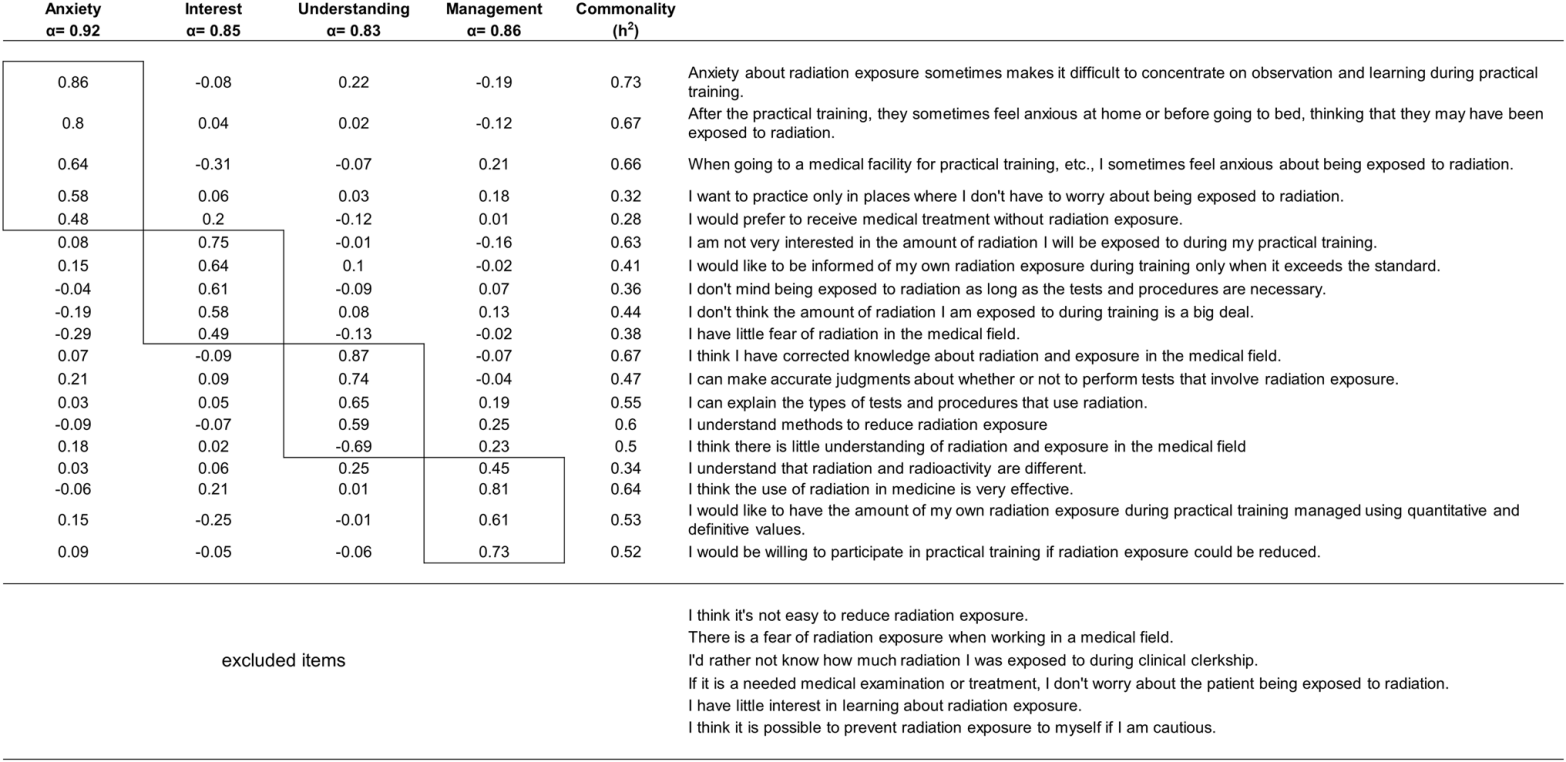
Factor loadings and communalities for items related to radiation exposure learning among medical students (*N* = 95)

“Anxiety” refers to vague fears regarding radiation exposure, “Interest” refers to the degree of curiosity or indifference toward radiation exposure, “Knowledge” refers to the self-perceived correctness of radiation-related knowledge, and “Management” refers to strategies and clarity in handling radiation exposure

All factor loadings ≥ |0.35| are shown. Cronbach’s alpha for each factor is indicated. Communalities (*h*^2^) are presented in the rightmost column

### Data collection

The participants were 95 fifth-year medical students (70 males, 25 females) who responded to both the pre-survey and the post-survey. They had completed their basic clinical medicine studies and passed the pre-Clinical Clerkship Objective Structured Clinical Examination (pre-CC OSCE) and CBT and were at the stage prior to participating in clinical practice. Informed consent was obtained from all participants prior to their participation, and the study protocol was approved by the Nara Medical University Ethics Committee. The anonymity of the participants was maintained throughout the study. Only students who responded to both surveys were included in the analysis.

### Data analysis

All statistical analyses were performed using R (The R Foundation for Statistical Computing, Vienna, Austria, version 4.0.3). The significance level was set at 5%, and a *p* value below 10% was considered indicative of a trend.

#### Exploratory factor analysis

To identify the underlying factor structure of the questionnaire items, an exploratory factor analysis was conducted on the 25 items. The number of factors was determined using eigenvalues, the scree plot, parallel analysis, and the MAP criterion. Maximum likelihood estimation was used for factor extraction, and Promax rotation was applied. The factor loading threshold was set at 0.35. Items that did not significantly load onto any single factor or loaded significantly onto multiple factors were excluded from further analysis.

#### Within-subjects design analysis of variance

To evaluate the impact of the lecture on the identified factor scores, 2 (survey period: pre-survey or post-survey) × (number of factors identified in the exploratory factor analysis) mixed-design analysis of variance was performed. Furthermore, similar mixed-design analyses of variance were conducted to examine the effects of gender and planned clinical training content (students who selected radiology or cardiology versus those who selected other departments) on the factor scores. Post hoc analyses using adjusted Bonferroni method were performed where appropriate to account for multiple comparisons.

## Results

### Exploratory factor analysis

First, in exploratory factor analysis, we conducted 25 items to clarify the factor structure of questions related to radiation exposure. In the procedure for exploratory factor analysis, the number of factors was estimated using the eigenvalues and the decay situation of the scree plot, parallel analysis, and MAP, and the four-factor structure that was most supported by each estimation method was adopted. Next, factor extraction was performed using the maximum likelihood method with Promax rotation. The criterion for factor loadings was set at 0.35, and items that did not contribute to any factor and items that contributed to multiple factors were excluded (6 items; Table1).

Factor 1: Anxiety about radiation exposure. This factor showed high loadings for items such as, “Anxiety about radiation exposure sometimes makes it difficult to concentrate on observation and learning during practical training” and “After practical training, I sometimes feel anxious at home or before going to bed, fearing possible radiation exposure”.

Factor 2: Interest in radiation exposure. This factor showed high loadings for items such as, “I am not very interested in the amount of radiation I will be exposed to during my practical training,” and “I would like to be informed of my own radiation exposure during training only when it exceeds the standard”.

Factor 3: Knowledge about radiation exposure. This factor showed high loadings for items such as, “I believe I have accurate knowledge about radiation and exposure in the medical field” and “I can accurately judge whether or not to perform tests involving radiation exposure”.

Factor 4: Management of radiation exposure. This factor showed high loadings for items such as, “I would like my radiation exposure during practical training to be managed using quantitative, definitive values” and “I believe that the use of radiation in medicine is highly effective”.

### Within-subjects design analysis of variance

The results of the mixed-design analysis of variance examining within subjects the effect of the lecture revealed significant main effects for survey period (pre vs. post) (*F*(1, 89) = 7.14, *p* < .0001, *η*^2^_*p*_ = .07) and factor (anxiety about radiation exposure, interest, knowledge, management) (*F*(3, 267) = 59.48, *p* < .0001, *η*^2^_*p*_ = .43). A significant interaction between survey period and factor was also observed (*F*(3, 267) = 2.27, *p* < .0001, *η*^2^_*p*_ = .03). Following up on the significant interaction with simple main effect tests for survey period within each factor, the results indicated that scores for “knowledge about radiation exposure” and “management of radiation exposure” were significantly higher in the post-survey than in the pre-survey (*p* < .001 for both, Table 2). Conversely, no significant change was observed in “interest in radiation exposure” and “anxiety about radiation exposure” between the pre- and post-surveys (Table 2).

**Table 2:**
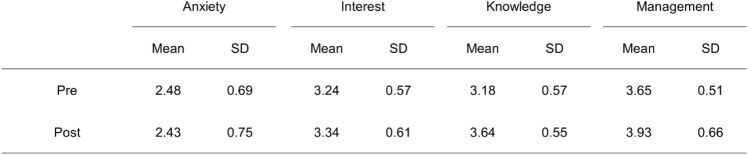
Mean and standard deviation (SD) of anxiety, interest, knowledge, and management scores for radiation exposure before (pre) and after (post) the lecture for all students

Higher scores indicate a greater degree of the measured factor. *Pre* before the lecture, *post* after the lecture. Scores are based on a five-point Likert scale

Furthermore, the maximum amount of radiation exposure allowed differs significantly between genders, with exposure to radiation for female students being strictly managed. To verify whether these factors also affect the students’ practical training, we conducted a mixed-design analysis of variance with 2 (gender: male or female) × 4 (factors: anxiety, interest, knowledge, and management of radiation exposure) using the difference of scores between post and pre (post–pre scores). Only the main effect of “factors” was significant (*F*(3, 267)=5.46, *p* < .0001, *η*^2^_*p*_ = .10), and there was no interaction effect (Table 3).

**Table 3:**
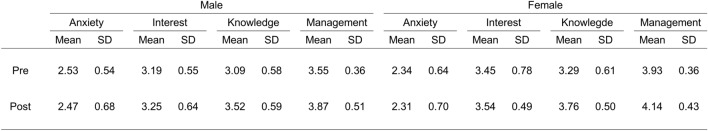
Average scores for anxiety, interest, knowledge, and management of radiation exposure in male vs. female students, before (pre) and after (post) the lecture

Male *n*=70, Female *n*=25. *Pre* before the lecture, *post* after the lecture. Scores are based on a five-point Likert scale

**Fig. 1:**
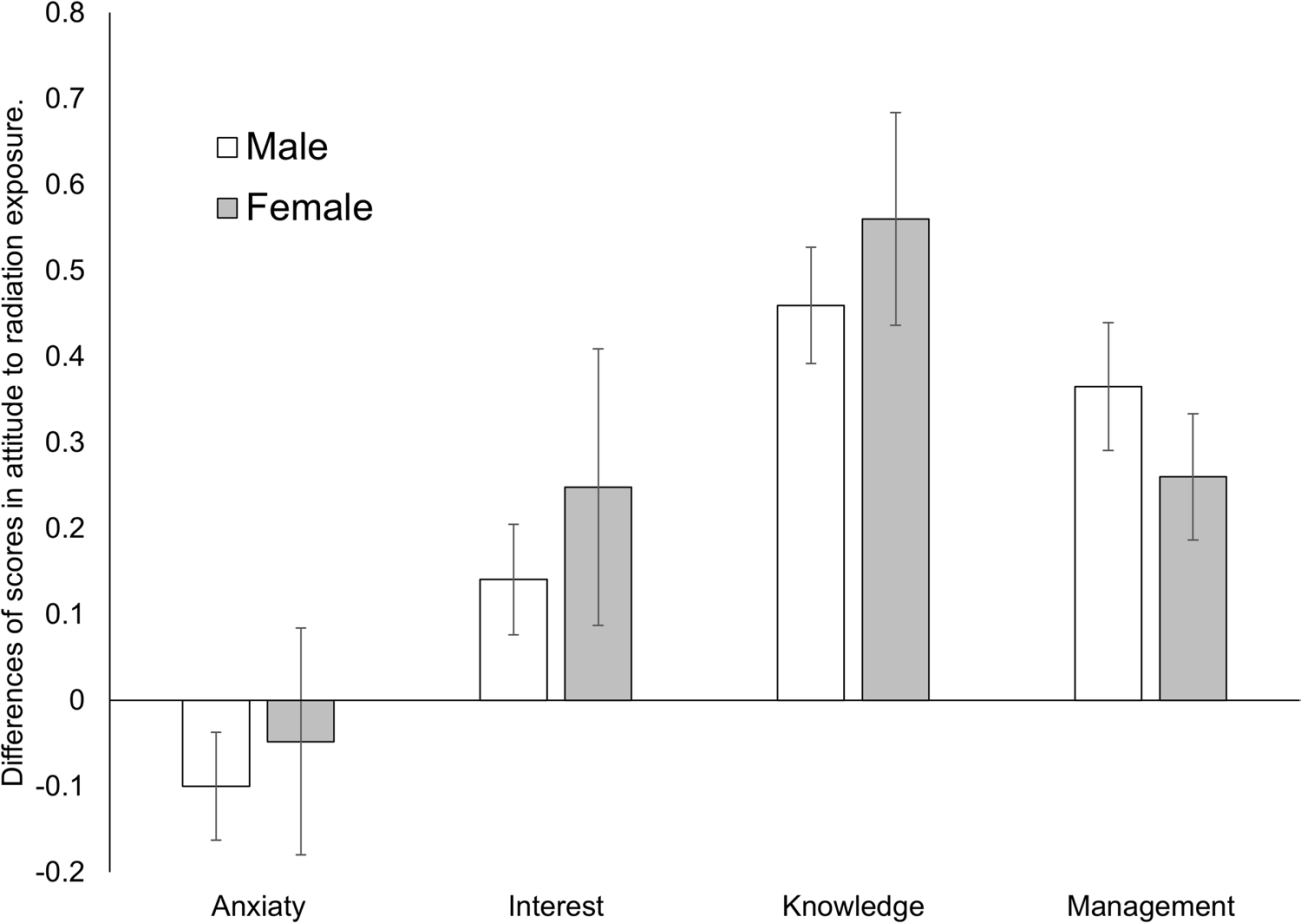
Differences (post–pre) in anxiety, interest, knowledge, and management of radiation exposure between the gender of students. Scores for anxiety decreased and scores for interest, knowledge, and management increased after the lecture for both males and females. Error bar indicates MSE (mean squared error)

In addition, since we used the difference of scores between post and pre, we tested whether the scores were significantly greater than zero for factors using the adjusted Bonferroni method (*p* < .05). As results, after the lecture, students showed significant increases in the scores for the other factors “interest in radiation exposure (*t*(89)= 2.07, *p* = .0205)”, “knowledge about radiation exposure (*t*(89)= 7.94, *p* < .0001)”, and “management of radiation exposure(*t*(89)= 5.31, *p* < .0001)”. (Figure 1).

**Fig. 2:**
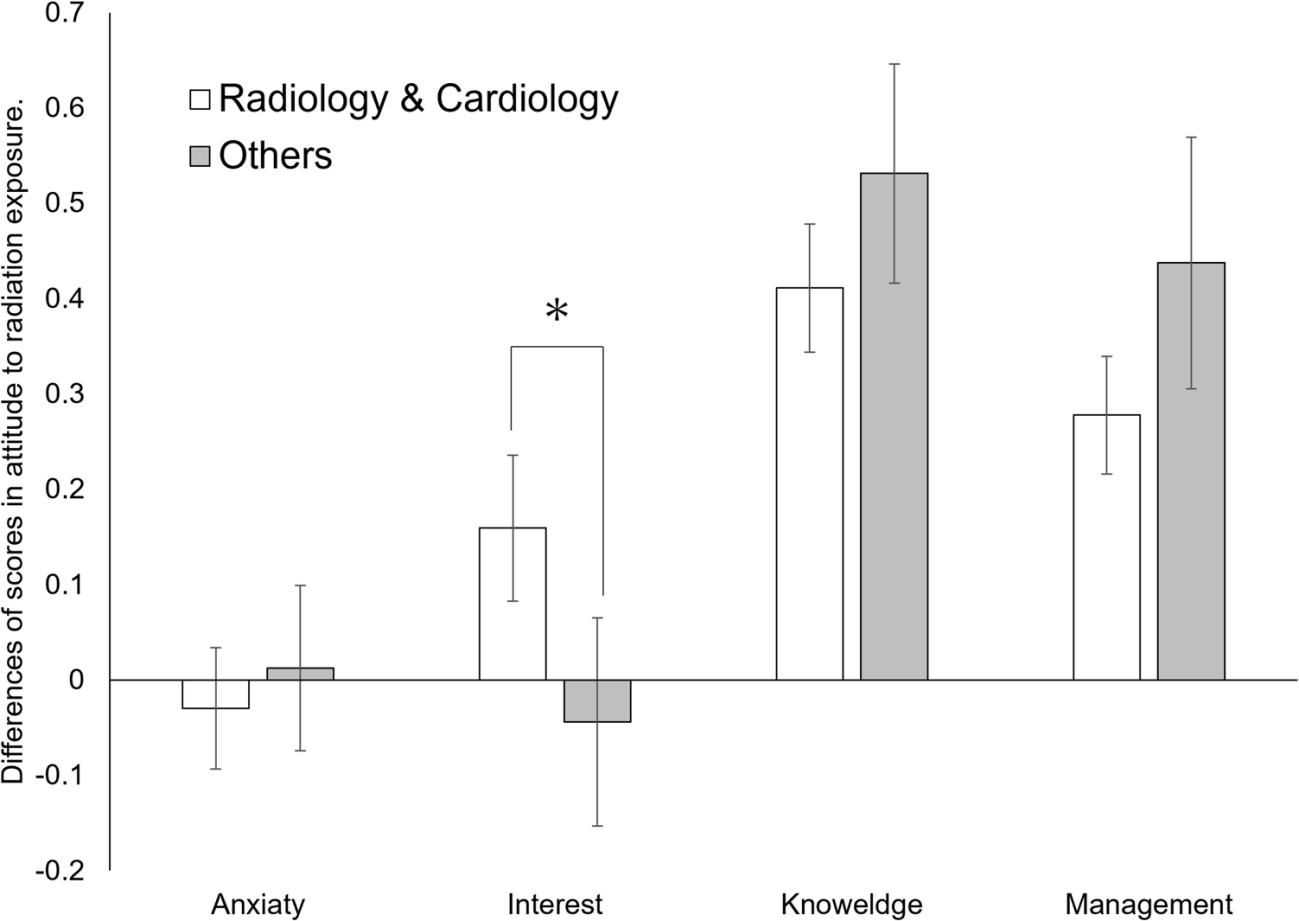
Differences (post–pre) in anxiety, interest, knowledge, and management of radiation exposure among students selecting radiology and cardiology vs. others. Comparing pre and post-lecture scores of interest increased for only students who selected radiology or cardiology. Radiology and cardiology n=57, others n=38. Pre = before the lecture; post = after the lecture. The asterisk (*) indicates a significant difference (*p* < .05). Error bar indicates MSE (mean squared error)

The mixed-design analysis of variance investigating the effect of clinical training selection using the difference in scores between post and pre (post–pre) revealed a significant interaction between training selection and factor (*F*(3, 267) = 3.36, *p* = .0309, *η*^2^_*p*_ = .02). Simple main effect tests showed a significant main effect of training selection for “knowledge about radiation exposure (*F*(1, 185) = 4.39, *p* = .0038, *η*^2^_*p*_ = .02)” and “management of radiation exposure (*F*(1, 185) = 6.34, *p* = .0013, *η*^2^_*p*_ = .03)” (Table 4). In addition, we tested whether the difference in scores was more significant than zero for each of the factors using the adjusted Bonferroni method (*p* <.05). The results showed that the scores for knowledge and management were significantly greater than zero regardless of whether the selection of department. The score for interest was a marginally significant trend (t(89)=2.07, *p* = .0853) only for students who selected radiology or cardiology (Figure 2).

**Table 4:**
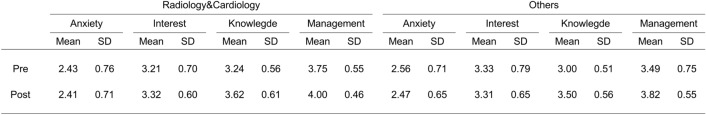
Average scores for anxiety, interest, knowledge, and management of radiation exposure in students selecting radiology and cardiology vs. others, before (pre) and after (post) the lecture

Radiology and cardiology *n*=57, others *n*=38. *Pre* before the lecture, *post* after the lecture. Scores are based on a five-point Likert scale

## Discussion

It is known that the Japanese population receives a high amount of medical radiation per capita and is very familiar with diagnostic imaging techniques, including CT and X-ray [17]. The entire population is presumably very aware of radiation exposure, owing to the damage caused by atomic bombs in World War II and the recent Fukushima nuclear power plant accident triggered by a tsunami; however, there is little concern about exposure in medical settings. This trend is the same in other countries as well, and despite the importance of radiation safety being recognized, research has consistently shown that there is a gap in the knowledge and awareness of medical students regarding radiation protection [5, 7, 18]. This situation can lead to medically dangerous behavior, and there is a possibility that it will increase the risk of unnecessary exposure for both students and patients [10, 19]. Although medical students have not previously been highly aware of radiation exposure issues [20], there have been multiple reports from countries such as Canada [21], Saudi Arabia [22], and Ethiopia [23] on the importance of education.

Similarly in Japan, considering the revision of the Medical Practitioners Act, it is important to reaffirm and educate medical students about radiation exposure and its risks to encourage their active participation in interventional radiology procedures. With reference to research from Australia [6] and the UK [8, 24], we decided to plan a lecture before the practical training, and then further consolidate the knowledge in the practical training. There have been reports that education using comics is useful [25], but there have also been reports that lecture-style education is effective enough on its own, so we did not use this method this time [26].

The lecture delivered just prior to clinical practice was intended to facilitate a smooth transition and reduce the likelihood of unnecessary radiation exposure incidents. The results of the analysis of the responses to our questionnaire showed that the scores for “knowledge about radiation exposure” and “management of radiation exposure” increased significantly before and after the lecture (both *p* < 0.001), indicating that the purpose of the lecture was achieved. On the other hand, no significant difference in students’ anxiety scores was observed before and after the lecture. A study of public health nurses in Japan [27] reported that lectures deepened students’ knowledge, which in fact reduced their anxiety about radiation exposure. Therefore, we expected that providing medical students with knowledge about radiation exposure through lectures would also reduce their anxiety, but the results were different from expectations. As reported in previous studies in China [28], the effectiveness of learning methods may vary depending on the knowledge level and background of learners, such as medical students or healthcare workers, as well as their learning and practical experience in the clinical workplace. We hypothesize that this may explain why our study showed different results from previous studies in Japan. In the future, we would like to further study teaching methods so that we can provide students with practical learning methods that can effectively reduce their anxiety.

In addition, in our research this time, we found no significant difference between males and females in any of the factors, including anxiety about exposure to radiation. In Japan, there are stronger restrictions on radiation protection for females than for males under the law. Both male and female students need to avoid radiation as much as possible, although the degree of strictness differs between them. Therefore, it is probably reasonable that no gender differences were found in the survey results.

Silvia Mamede & Henk G Schmidt [29] have stated in their article that when discussing educational effectiveness, increasing interest in a subject can lead to self-reflection and further sustained and self-motivated learning, and is therefore extremely important. Improving “interest” is therefore considered to be the most important educational outcome in radiation exposure as well, but in our study, no significant increase was demonstrated by the lectures. It is possible that education using only lecture-style teaching methods made it difficult to specifically imagine real medical situations, and that this limited the ability to deepen students’ interest. In future education, it is thought to be important to incorporate methods that encourage students to participate more actively in learning, such as through simulation-based education, and to promote reflection on practice, thereby further increasing interest. In addition, the fact that there was no improvement in interest among students who had selected departments where the risk of radiation exposure was thought to be low is thought to be because they did not directly associate their knowledge of radiation with their future field of professional specialization. The practical knowledge of radiation protection is essential for all medical professionals in the future, no matter which medical department they advance to. Therefore, we, as educators, need to convey this point clearly to students by effectively combining lectures and clinical practice, and this is precisely the important point in teaching students about radiation exposure.

One limitation of our study is that it is a single-year investigation involving only medical students at one university, resulting in a small sample size given the significant changes in their training. In Japan, the Medical Practitioners’ Act has been revised for the first time in 75 years, and clinical clerkships are now being changed from the observation-based training that had been the norm in the past to clinical clerkships in which students serve as “student doctors” and become members of the medical team. Naturally, there are likely to be more opportunities for students to be exposed to radiation during clinical clerkships. Against this background, we thought that it would be meaningful to teach medical students about radiation exposure and to have them experience it before their training. However, based on the results of this study, it appears that lectures alone are not effective enough. It is not yet known within the scope of this study what more effective learning methods are for medical students.

Our current study was based on students’ own “self-assessment” of their knowledge of radiation and specific protection methods, and it was not possible to measure their actual understanding and skills. In the future, it will be necessary to verify what educational methods would be more effective by adding educational evaluations not only through lectures, but also through practical training, such as the introduction of on-site practical tests, OSCE-style evaluations, and measurement of knowledge retention after all practical training is completed. For students to understand the significance of learning about radiation exposure, it would be important to repeat the process of implementation and feedback, for example, by lending digital dosimeters to students and visualizing their individual radiation exposure during clinical clerkship, so that they can realize the effectiveness of the radiation exposure reduction they have learned.

## Conclusion

Lectures on radiation exposure for senior medical students significantly increased their knowledge and scores on radiation protection. Increasing interest in radiation exposure proved challenging among students who chose departments perceived to have a low likelihood of exposure during clinical training, and developing effective educational methods for these students remains a future challenge. The results of this study highlight the importance of radiation safety education for medical students about to begin their clinical clerkships and suggest areas for improvement in educational content and methods.
